# Graphical coding data and operational guidance for implementation or modification of a LabVIEW®-based pHstat system for the cultivation of microalgae

**DOI:** 10.1016/j.dib.2017.04.046

**Published:** 2017-04-29

**Authors:** Rachel L. Golda, Mark D. Golda, Tawnya D. Peterson, Joseph A. Needoba

**Affiliations:** a Institute of Environmental Health, Oregon Health & Science University, 3181 SW Sam Jackson Park Road, Portland, OR 97239-3098, United States; b Science and Technology Center for Coastal Margin Observation and Prediction, Oregon Health & Science University, 3181 SW Sam Jackson Park Road, Portland, OR 97239-3098, United States; c SSI Consulting, P.O. Box 2155, Shelton, WA 98584, United States

**Keywords:** PHstat, Chemostat, Ocean acidification, LabVIEW®, Phytoplankton culture

## Abstract

The influence of pH on phytoplankton physiology is an important facet of the body of research on ocean acidification. We provide data developed during the design and implementation of a novel pHstat system capable of maintaining both static and dynamic pH environments in a laboratory setting. These data both help improve functionality of the system, and provide specific coding blocks for controlling the pHstat using a LabVIEW® virtual instrument (VI). The data in this paper support the research article “Development of an economical, autonomous pHstat system for culturing phytoplankton under steady state or dynamic conditions” (Golda et al. [2]). These data will be of interest to researchers studying the effects of changing pH on phytoplankton in a laboratory context, and to those desiring to build their own pHstat system(s). These data can also be used to facilitate modification of the pHstat system to control salinity, temperature, or other environmental factors.

**Specifications table**

**Table:** 

Subject area	*Biology, Environmental Science*
More specific subject area	*pHstat and phytoplankton culture systems*
Type of data	*Data from graphical code development, supplementary design details from* Golda et al. [2], *to aid pHstat design*
How data was acquired	*Data was acquired using a LabVIEW® virtual instrument to design programs to monitor, modify, and record pH data*
Data format	*Schematics, code*
Experimental factors	*The graphical coding language LabVIEW® was used to create a virtual instrument for running a pHstat system*
Experimental features	*The authors designed, built, and tested a pHstat system for the long-term growth of phytoplankton under steady-state or variable pH conditions*
Data source location	*Not applicable; laboratory based study*
Data accessibility	*Data are available in this article*

**Value of the data**

• The technical (i.e., design-related) graphical coding blocks presented here provide data regarding system specifications with which an independent research easily replicate a phytoplankton culture system capable of maintaining stable (steady state) and dynamic pH levels.
• The coding data provided can be used to extend the current capabilities of chemostats to incorporate sensor-driven monitoring of conditions such as pH (as in this case), salinity, nutrients, or turbidity to investigate responses by phytoplankton to a broad suite of environmental conditions in steady state or dynamic modes.
• These data are important for researchers or groups developing steady-state or dynamic systems with long-term applications. They are also important for researchers examining phytoplankton physiology as it relates to pH and ocean acidification

## 1 Data

**Fig. 1:**
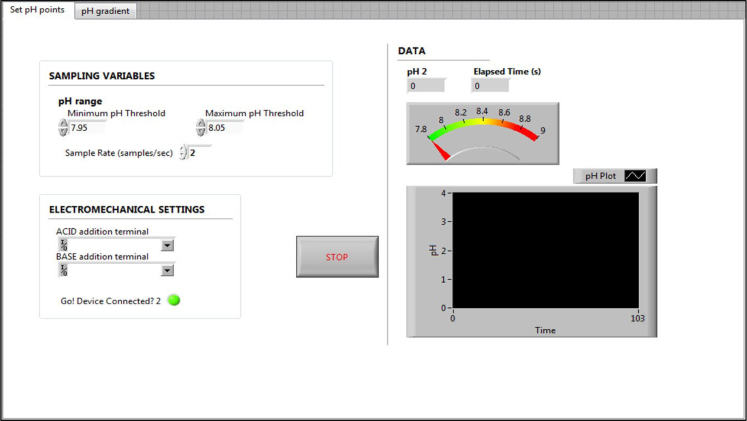
Front panel of virtual instrument (VI) for producing a steady state pH environment.

**Fig. 2:**
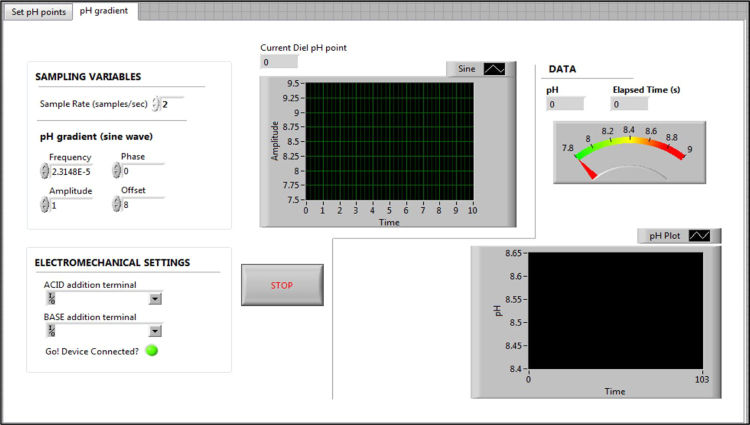
Front panel of virtual instrument (VI) for producing a dynamic pH environment based on a sine wave.

**Fig. 3:**
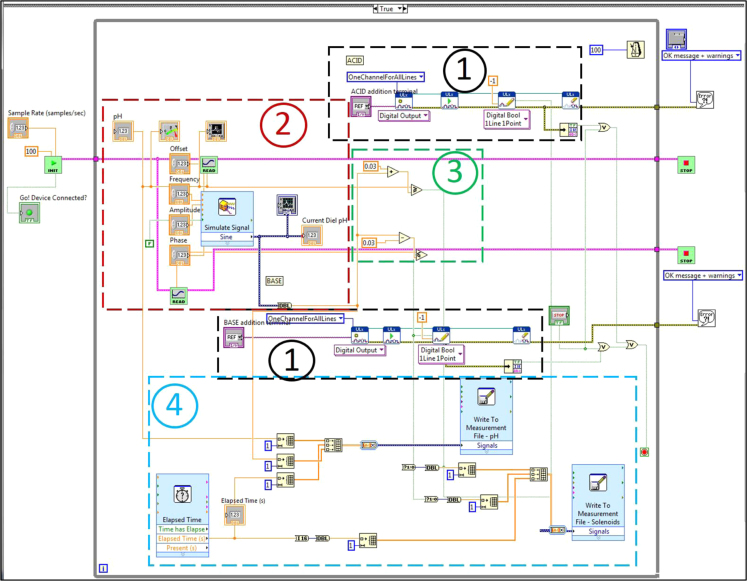
Block diagram for VI showing graphical code for dynamic pH functionality. Sections 1, 2, 3, and 4 correspond to Fig. 3.1, Fig. 3.2, Fig. 3.3, Fig. 3.4, respectively.

**Fig. 4:**
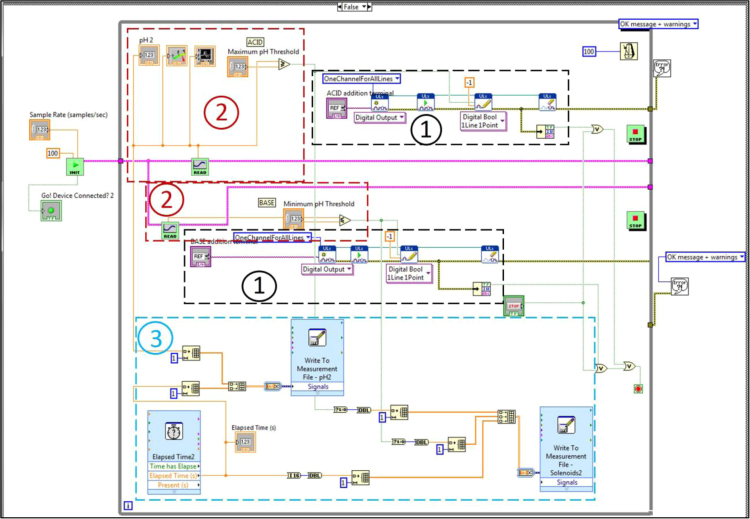
Block diagram for VI showing graphical code for steady state pH functionality. Sections 1, 2, and 3 match up to Fig. 4.1, Fig. 4.2, Fig. 4.3, respectively.

The front panel and block diagrams constituting the LabVIEW® Virtual Instrument (VI) are shown (Fig. 1, Fig. 2, Fig. 3, Fig. 4) to demonstrate the software/programmatic component of the pHstat system. Fig. 1, Fig. 2 depict the front panel of the two functionalities of the VI. The front panel is the portion of the VI with which the user interacts during an experiment or whenever the VI is running.

**Fig. 3.1:**
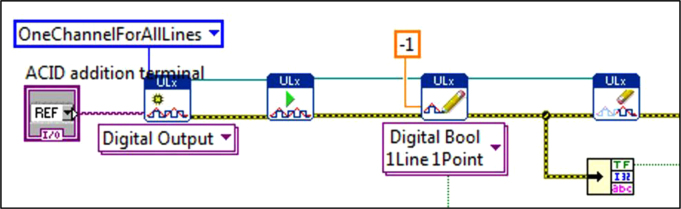
Section 1 from Fig. 3 expanded to show programmatic detail. Depicts graphical code for connecting the MC control board to the pHstat system.

**Fig. 3.2:**
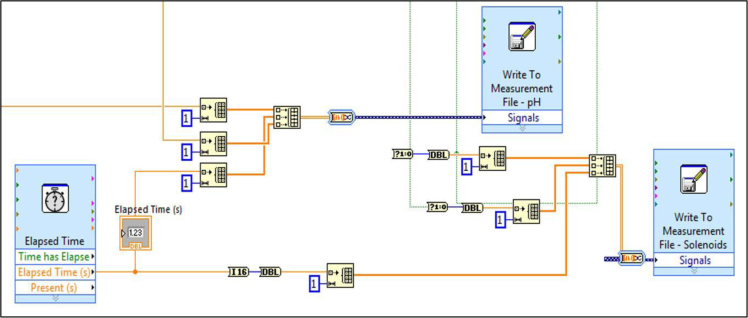
Section 2 from Fig. 3 expanded to show programmatic detail. Depicts graphical code for generating and recording data from the subVIs “Elapsed Time,” “Write to Measurement File – pH,” and “Write to Measurement File – Solenoids.” Golda et al. [2] provides details on the data saved.

**Fig. 3.3:**
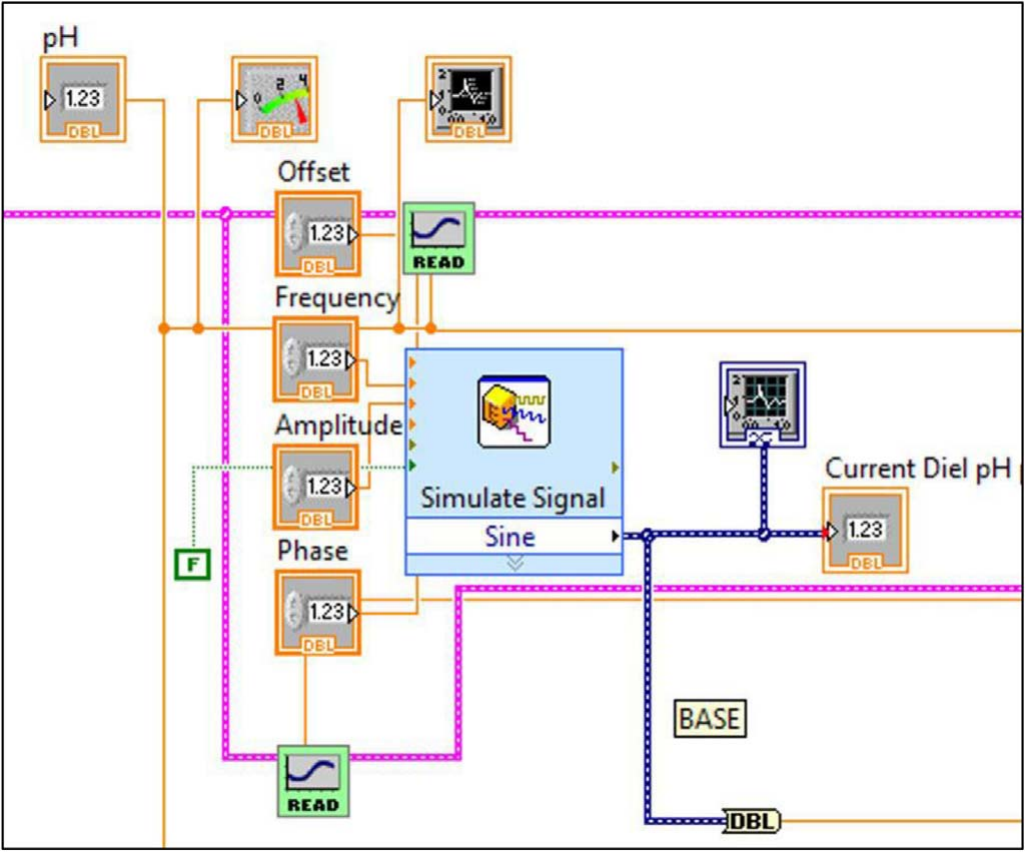
Section 3 from Fig. 3 expanded to show programmatic detail. Shows subVI for providing the guide pH for the dynamic functionality of the pHstat. Guide pH is generated using a sine wave, with the user setting the frequency, offset, amplitude, and phase in order to time the wave to start at a desired pH. Details on how the dynamic functionality works can be found in Golda et al. [2].

**Fig. 3.4:**
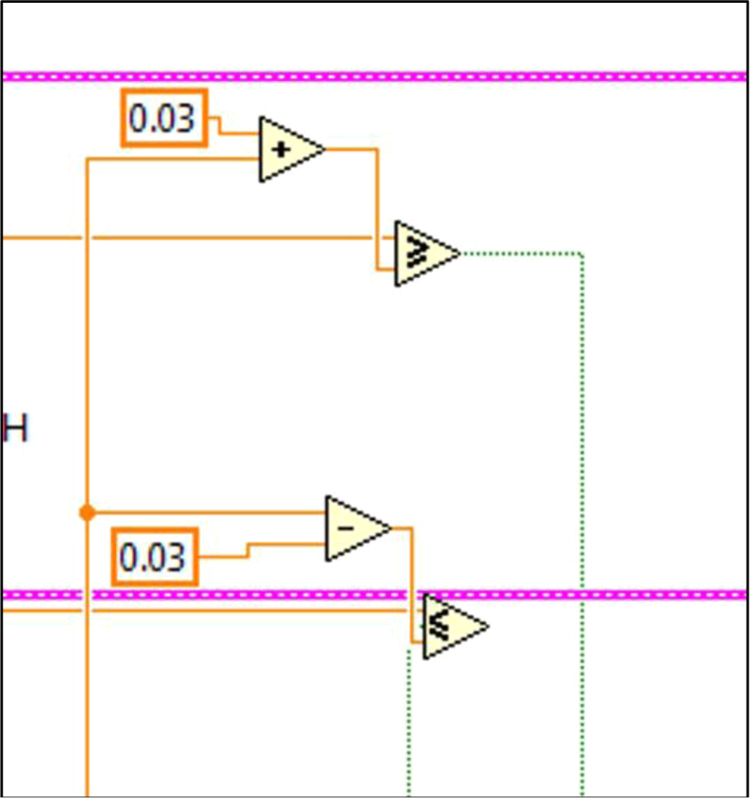
Section 4 from Fig. 3 expanded to show programmatic detail. Depicts programmatic element of pH control using Boolean operators to maintain pH thresholds within a set pH points of the guide pH at any given time.

**Fig. 4.1:**
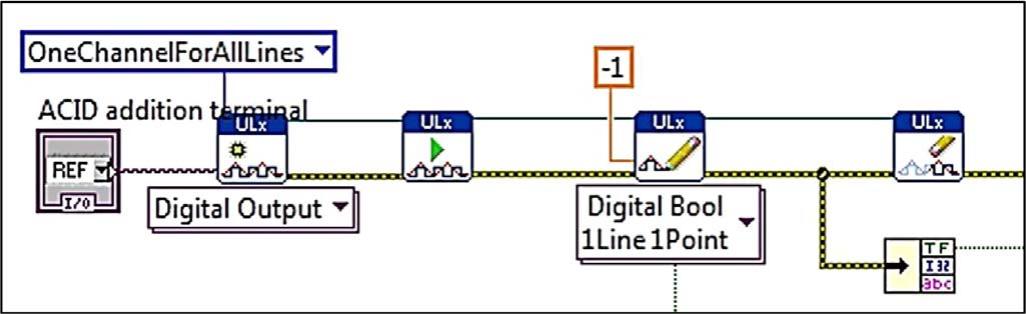
Section 1 from Fig. 4 expanded to show programmatic detail. Depicts graphical code for connecting the MC control board to the pHstat system.

**Fig. 4.2:**
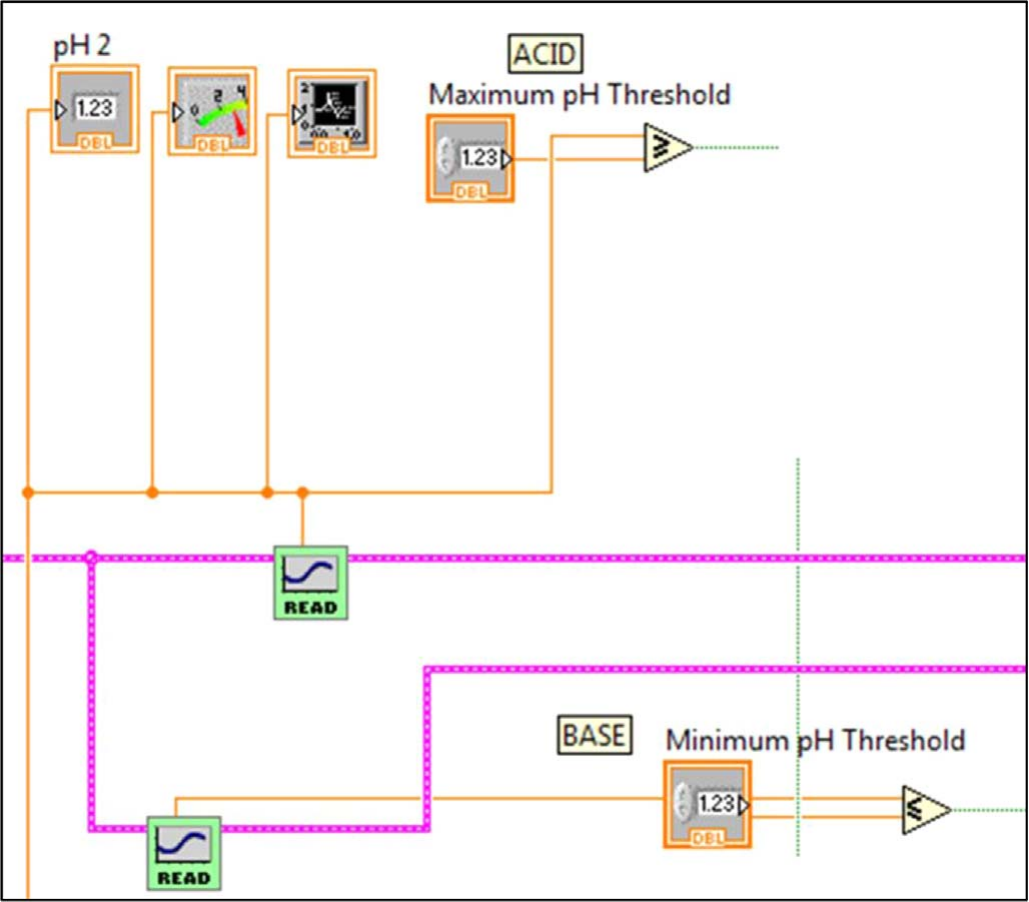
Section 2 from Fig. 4 expanded to show programmatic detail. Depicts programmatic element of pH control using front panel indicators to maintain pH within thresholds. Broken wires connect to other elements, which were deleted for clarity.

**Fig. 4.3:**
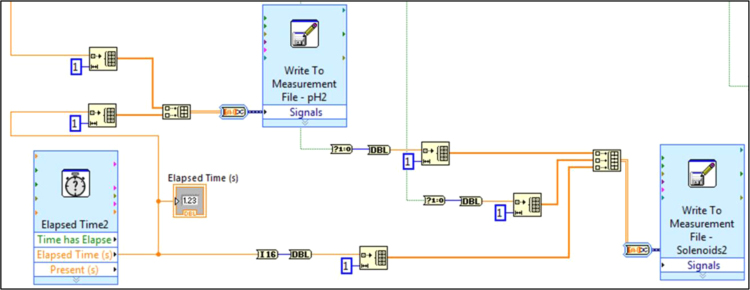
Section 3 from Fig. 4 expanded to show programmatic detail. Depicts graphical code for generating and recording data from the subVIs “Elapsed Time,” “Write to Measurement File – pH,” and “Write to Measurement File – Solenoids.” Golda et al. [2] provides details on the data saved.

The block diagrams contain the actual graphical code for running the LabVIEW® VI (Fig. 3, Fig. 4). Figs. 3.1–3.4 and 4.1–4.3 show the graphical code blocks separated broadly by function. Details on connecting this code language to the electromechanical portion of the pHstat can be found in Golda et al. [2]. The figures are followed by operational instructions for using the VI as it operates according to the coding blocks presented in Fig. 3, Fig. 4, Fig. 3.1, Fig. 3.2, Fig. 3.3, Fig. 3.4, Fig. 4.1, Fig. 4.2, Fig. 4.3, 3.1–3.4, and 4.1–4.3.

Fig. S1 shows the detailed schematic of the electromechanical portion of the pHstat system, the conditioning electronically operated relay grouping (GEORG).

## 2 Experimental design, materials and methods

In brief, the pHstat includes all of the components necessary for high-resolution, in situ pH monitoring and autonomous pH adjustments. The pH of the culture vessel is continuously monitored by an in situ pH sensor (Vernier, Beaverton, OR) interfaced to a LabVIEW® Virtual Instrument (VI) via a series of electromechanical components. LabVIEW® is a graphical coding language [1] designed by National Instruments (Austin, TX). Our VI uses maximum and minimum pH thresholds determine when to add liquid reagents or gasses to the culture vessel in order to modify the pH. When the pH returns to the acceptable thresholds, the system deactivates and remains on standby until the next pH deviation occurs. A complete description of the system can be found in Golda et al. [2].

## Appendix A Supplementary material

Supplementary material Fig. S1 Schematic of GEORG, with wiring diagram for DB15 (D-subminiature, 15 pin) connectors mounted on the box. A and B indicate DB15 connectors that provide signal transfer to and from GEORG, respectively. A is a male DB15 that receives signals from the computer; B is a female DB15 that transmits signals to the pH modification manifolds. Connector cables from the digital input/output device and to the pH-modification manifolds must be customized to match these DB15 connectors. Red lines indicate power supply wires, bold black lines indicate ground wires, thin black lines indicate signal wires running from the computer.
