# Supplementary material for: Graphical coding data and operational guidance for implementation or modification of a LabVIEW®-based pHstat system for the cultivation of microalgae
Source: Data Brief. 2017 Apr 29;12:463–70. doi: 10.1016/j.dib.2017.04.046 (PMC5423297; doi:10.1016/j.dib.2017.04.046)
Supplement: Supplementary file 2 — Supplementary material Fig. S1 Schematic of GEORG, with wiring diagram for DB15 (D-subminiature, 15 pin) connectors mounted on the box. A and B indicate DB15 connectors that provide signal transfer to and from GEORG, respectively. A is a male DB15 that receives signals from the computer; B is a female DB15 that transmits signals to the pH modification manifolds. Connector cables from the digital input/output device and to the pH-modification manifolds must be customized to match these DB15 connectors. Red lines indicate power supply wires, bold black lines indicate ground wires, thin black lines indicate signal wires running from the computer. [file mmc2.docx]

**Supplementary material**

*Operation of the VI*

This section describes how to operate the virtual instrument (VI) that runs the pHstat system. We recommend that the user disable the “Sleep” and “Hibernate” settings on their computer when using the VI, as these functions interrupt VI continuity.

1. Setting up the system

IMPORTANT: Due to the risk of electrical shock, whenever the user is setting up or handling the electronic or electromechanical portions of this system, they should ensure that the system is not connected to any power supply. This includes computer connections and wall sockets. All connections to power sources should be made after the user has completed hands-on work with the system.

1. Install drivers and necessary software for pH probe and digital input/output (I/O) device.
2. Connect the pH probe to the computer (if using a Go!Link (Vernier), connect the probe to the Go!Link, then connect the Go!Link to the computer).
3. Connect the relay array (GEORG) to the digital I/O device by inserting the appropriate wires into the screw terminals of the I/O device. A pinout diagram of the screw terminals should be available in the manual for the I/O device. We used a USB-1024LS digital I/O device (Measurement Computing Corporation, Norton, MA) as an interface between the VI and the relay array. NOTE: This device is sensitive to electrostatic discharge (ESD). We suggest the operator grounds himself/herself prior to touching this device.
4. Connect GEORG to the pH modification manifolds using the custom input/output cables. Make sure that each relay for GEORG is connected to the appropriate solenoid valve on the modification manifold. Use Figure 3 for relay orientation.
5. Connect the digital input/output device to the computer using the provided universal serial bus (USB) connector cable.
6. Connect GEORG’s power supply to the wall.
7. Operating the VI during an experiment
8. Complete setup steps detailed in section A of this appendix.
9. ELECTROMECHANICAL SETTINGS
   1. These terminals correspond to the screw terminals chosen to activate the acid/base solenoids on the pH modification manifolds.
   2. Use the pinout diagram associated with the digital I/O device to choose the appropriate terminals.
10. SAMPLING VARIABLES
    1. Select VI functionality (front panel). Default setting is Steady-state; press button labeled “pH Gradient (ON/OFF)” to switch to a dynamic pH regime.
    2. Use tabs at top of screen to set parameters for pH regime.
    3. FOR STEADY-STATE PH
       1. Set minimum pH threshold.
       2. Set maximum pH threshold.
       3. Set sample rate. NOTE: If using the reagent manifold do not set the sample rate for more than 2 samples per second; this will cause the system to overcompensate and result in loss of pH control.
    4. FOR DYNAMIC PH
       1. Set sample rate.
       2. Set parameters for guide pH, “pH gradient (sine wave),” including frequency, amplitude and offset (mid-point pH).
11. Press “Run” button (arrow at top left corner).
12. Save data when prompted. There will be two save prompts; one is for the pH data, the other is for the solenoid activity data. If the user doesn’t want to save their data click “Cancel” for each save prompt and confirm “Do not save.”
13. To stop the experiment, press the “STOP” button.
14. Real-time data is shown on the right side of the screen. pH data is displayed as a numerical value, on a scale meter and as a waveform chart. Elapsed time is given in seconds. Solenoid activity data is not displayed, and must be accessed directly from the saved data.
15. Data is saved as a .lvm file.
16. Data Processing
17. Data is saved as a .lvm file.
18. This file type can be opened using Microsoft® Excel or Notepad.
19. Data is given in column format. The first column is blank for both data types.
20. For the pH data sets, column 2 is pH data, column 3 is elapsed time in seconds.

For the solenoid activity data, column 2 is the activity data for the acid-dispensing solenoid, column 3 is the activity data for the base-dispensing solenoid, and column 4 is the elapsed time in seconds.


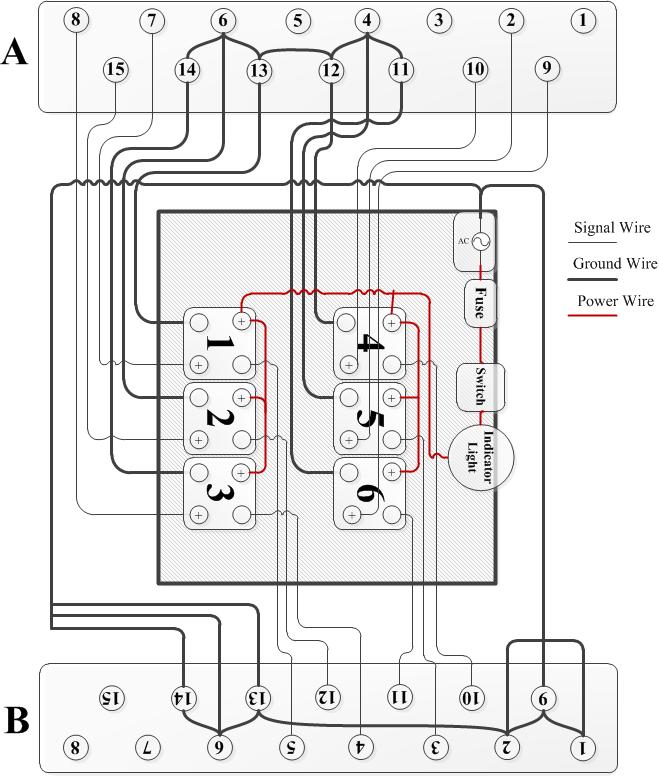


Fig. S1: Schematic of GEORG, with wiring diagram for DB15 (D-subminiature, 15 pin) connectors mounted on the box. A and B indicate DB15 connectors that provide signal transfer to and from GEORG, respectively. A is a male DB15 that receives signals from the computer; B is a female DB15 that transmits signals to the pH modification manifolds. Connector cables from the digital input/output device and to the pH-modification manifolds must be customized to match these DB15 connectors. Red lines indicate power supply wires, bold black lines indicate ground wires, thin black lines indicate signal wires running from the computer.
